# Point-of-care CAR-T cell manufacturing as a path to equity in cancer immunotherapy in a middle-income country

**DOI:** 10.1016/j.omton.2026.201290

**Published:** 2026-07-21

**Authors:** Ha-My Cao, Michael Aigner

**Affiliations:** 1 Hanoi Medical University, No. 1 Ton That Tung Street, Kim Lien, Hanoi 10000, Vietnam; 2 National Institution of Hematology and Blood Transfusion, No. 5 Pham Van Bach Street, Cau Giay, Hanoi 10000, Vietnam; 3 Department of Internal Medicine 5 - Hematology and Oncology, Friedrich-Alexander-Universität Erlangen-Nürnberg and Universitätsklinikum Erlangen, Erlangen, Germany; 4 Bayerisches Zentrum für Krebsforschung (BZKF), Erlangen, Germany; 5 Deutsches Zentrum Immuntherapie (DZI), Friedrich-Alexander-Universität Erlangen-Nürnberg and Universitätsklinikum Erlangen, Erlangen, Germany

## Main Text

In the previous issue of Molecular Therapy Oncology, Nguyen and colleagues presented a study that represents an important milestone for Vietnam and for global efforts to expand access to CAR T cell therapy in low- and middle-income countries.^1^ CAR T cell therapy targeting CD19 has become a key option for patients with relapsed or refractory B cell acute lymphoblastic leukemia (ALL) and non-Hodgkin lymphoma (NHL), who otherwise have very poor response with chemotherapy.^2^^,^^3^ This phase 1, single-center trial, run by a private medical center in Vietnam, had tested point-of-care-manufactured CD19 CAR T cells (VinCART) in 16 patients (8 ALL and 8 NHL) who had typically received three prior lines of therapy. The cohort reflects real-world high-risk disease: all eight patients with ALL had relapsed disease (half refractory), and three of eight patients with NHL were refractory. Patients with ALL achieved 100% complete remission (CR) at day 30; three later relapsed, giving a 1-year progression-free survival (PFS) of 62.5%, while the NHL group sustained CR in 7 out of 8 patients with 1-year PFS of 87.5% and only one progression-related death. Toxicity was manageable, with cytokine release syndrome occurring in most patients but rarely exceeding grade two.

Despite the high-risk profile, the clinical outcomes were encouraging, suggesting that this locally manufactured product can deliver outcomes comparable to international experience in a Vietnamese setting and that, based on the existing experiences in cellular therapeutic approaches such as stem cell transplantation, Vietnamese centers can manage CAR T-related toxicities effectively.^4^ The study also shows the challenge of prolonged B cell aplasia and hypogammaglobulinemia, which occurred in the majority of patients. Safety is critical in a resource-limited context such as that in Vietnam since complications would result in extended hospitalization and increased financial burden. This has important implications for long-term supportive care, particularly in settings where intravenous immunoglobulin is costly and not routinely reimbursed. Nevertheless, the overall safety profile remained acceptable and consistent with global experience.

One of the most impressive achievements is the demonstration that high-quality CAR T cell products can be produced locally using the CliniMACS Prodigy platform. The median vein-to-vein time of up to 13 days is substantially shorter than the three to six weeks typical of centralized commercial manufacturing. All infused products met stringent release criteria, including high viability, consistent CAR expression, robust cytotoxic potency, and acceptable vector copy numbers. These results confirm existing data that point-of-care manufacturing can achieve product quality comparable to centralized facilities in the United States or Europe.^5^^,^^6^^,^^7^

Economically, the total per-patient cost was about USD 120,000, of which USD 80,000 was allocated for cell manufacturing and USD 40,000 for clinical care—far below typical commercial CAR T prices in high-income countries. The Vietnamese manufacturing cost structure is similar to other middle-income countries such as India (USD 50,000) or other academic-based, decentralized CAR T cell programs, such as in Spain (€90,000).^8^^,^^9^ Two-thirds of the clinical costs were inpatient expense, suggesting that further savings may be achievable with safer outpatient models—which is already being implemented in some Western centers—and better complication control.^10^^,^^11^ Together, these data indicate that point-of-care CAR T in Vietnam can offer good remission rates, acceptable toxicity, and improved access compared with imported commercial products, while highlighting the need to manage relapse risk (especially CD19-negative disease) and long-term immune suppression.

Beyond the clinical and manufacturing results, the broader implications for low- and middle-income countries are substantial. Vietnam’s healthcare system is characterized by a centralized model of care, in which specialized tertiary centers play a pivotal role alongside general hospitals in managing complex diseases. For example, a public tertiary referral hospital could receive approximately 400 newly diagnosed cases of NHL per year and 300 cases of ALL (internal/unpublished data). With such volume of patients, the demand for treatment is immensely high. Stem cell transplantation has become available since the 2010s,^4^ and several targeted agents have been introduced in the last five years but not fully reimbursed. However, novel agents for acute leukemia (e.g., blinatumomab, inotuzumab, gemtuzumab ozogamicin, etc.), bispecific antibodies, and commercial CAR T cell products remain out of reach, which poses challenges to refractory disease. This is primarily driven by a lack of commercial profitability and the prolonged, cumbersome regulatory approval processes that deter foreign pharmaceutical investments.

Point-of-care CAR T cell manufacturing directly addresses several of these challenges by enabling local production, reducing cost, and shortening delivery times. Vietnam’s existing experience with stem cell processing provided a strong foundation for CAR T cell manufacturing, and many other middle-income countries possess similar infrastructure within national cancer centers or blood banks. The study demonstrates that rigorous aseptic technique, quality control, and batch release procedures can be implemented successfully at least in highly specialized centers. Therefore, in these settings, a decentralized manufacturing with local hubs at large medical institutions such as tertiary hospitals or universities is one potential approach to enhance patient access. More widespread distribution to smaller or even local sites does not seem to be advisable at the moment, as requirements for successful implementation are still very high.

Importantly, point-of-care manufacturing reduces dependence on commercial markets and allows local institutions to prioritize diseases prevalence in their region, control pricing, and adapt manufacturing protocols to local constraints. This autonomy is increasingly valuable as the global cell and gene therapy landscape undergoes rapid consolidation and strategic shifts. Moreover, point-of-care platforms provide a flexible infrastructure that can accommodate next-generation therapies, including *in vivo* CAR T cell technologies, allogeneic products, and CAR T cell applications in autoimmune diseases.

In summary, the work of Nguyen and colleagues demonstrates that point-of-care CAR T cell manufacturing is feasible, safe, and clinically effective in a middle-income country. Their experience offers a blueprint for expanding CAR T cell access across low- and middle-income regions and highlights the transformative potential of decentralized manufacturing. With strategic investment in infrastructure, workforce development, and quality systems, countries such as Vietnam can not only adopt but also innovate within the CAR T cell field, ensuring that advanced immunotherapies become accessible to patients who previously had no realistic path to receiving them.

## Declaration of interests

M.A. received travel support and speakers’ honoraria from Miltenyi.
